# A rapid review of menopausal education programmes

**DOI:** 10.1007/s00737-024-01476-8

**Published:** 2024-05-25

**Authors:** Claire McFeeters, Katy Pedlow, Deborah McGinn, Karen McConnell

**Affiliations:** 1 School of Health Sciences, Ulster University, Belfast, Northern Ireland; 2 Informing Choices, Londonderry, Northern Ireland; 3 School of Nursing and Midwifery, Queens University Belfast, Belfast, Northern Ireland

**Keywords:** Menopause, Education, Rapid review

## Abstract

**Introduction:**

Menopause is a significant life event that can impact a woman's quality of life and mental health due to hormonal changes. Menopause education programmes play a crucial role in increasing awareness and knowledge about menopause in women. This rapid review aimed to identify the structure and components of menopause education programmes and summarise the evidence of their effectiveness in improving menopausal knowledge, symptoms, and quality of life.

**Methods:**

The Cochrane rapid review methodology was employed, involving systematic searches in four databases. The eligibility criteria included primary research on menopause education programmes for adults, and studies reporting menopause-related outcomes.

**Results:**

A total of 39 studies were included in the review, with most (*n* = 26/39, 66.7%) published in the last decade. The majority of interventions were delivered in group settings, providing advantages such as a supportive environment and shared experiences among participants. The most frequently covered topics included signs and symptoms of menopause, treatment/management, and lifestyle factors. The review identified evidence of effectiveness in supporting menopause education programmes for improving women's knowledge, symptoms, and quality of life. However, inconsistent reporting of intervention components hindered replication and implementation.

**Conclusion:**

The review suggests the need for comprehensive reporting of interventions, and inclusion of premenopausal women, and recommends that future menopause education interventions are inclusive for all ages and abilities. Overall, studies included in this review support the use of menopause education programmes for improving women's understanding and management of menopause.

**Supplementary information:**

The online version contains supplementary material available at 10.1007/s00737-024-01476-8.

## Introduction

Menopause is the permanent cessation of menstrual cycles for 12 consecutive months (World Health Organization 1996) that typically occurs between the ages of 45–55 (World Health Organization 2022). It involves changes in hormone levels, particularly a decrease in estrogen and progesterone, which can significantly impact both physiological and psychological aspects of quality of life (Woods and Utian 2018; Ye et al. 2022). According to NICE guidance on the diagnosis and management of menopause (National Institute for Health and Care Excellence 2015), menopausal women and their families or spouses should be provided with advice and information regarding the stages, symptoms, management, treatment benefits and risks, as well as long-term health implications associated with this transition.

Menopause education programmes can play a vital role in increasing awareness, improving attitudes, reducing impact on mental health and enhancing the level of information and knowledge about this phase (Golyan Tehrani et al. 2007). In addition to enhancing knowledge and symptom management, education programmes are beneficial in addressing psychological symptoms associated, such as mood swings and anxiety (Holloway 2022). Various educational approaches have been successfully employed using different modes of delivery including group (Gebretatyos et al. 2020; Li et al. 2023; Liao and Hunter 1998) and individual education sessions (Ahmady et al. 2022). Furthermore, a plethora of educational methods have been used such as written information (Gebretatyos et al. 2020; Li et al. 2023; Liao and Hunter 1998), face-to-face presentations (Gebretatyos et al. 2020; Li et al. 2023), group discussions (Gebretatyos et al. 2020; Li et al. 2023; Liao and Hunter 1998) and self-directed resources (Ahmady et al. 2022). Research evaluating the effectiveness of education interventions has suggested that they can enhance knowledge of menopause symptoms (Ahmady et al. 2022; Keye et al. 2023) associated such as weight gain, increase in facial hair, menstrual cycle irregularity, skin dryness, urinary frequency, dysuria, insomnia, depression and hot flashes (Holloway 2022; Gebretatyos et al. 2020; Li et al. 2023) and quality of life covering vasomotor, psychosocial, physical and sexual domains (Shobeiri et al. 2017) in individuals. In addition, these programmes can empower individuals to effectively manage the signs and symptoms of menopause when they arise (Yazdkhasti et al. 2015). Whilst a recent scoping review (Macpherson and Quinton 2022) summarised the evidence related to education of healthcare professionals, to date, no review has synthesized the literature on education of pre, peri, and postmenopausal women. To enable delivery of menopausal education programmes within a range of contexts it is important to identify what constitutes an effective intervention in terms of educational and clinical outcomes for women.

This review forms part of a larger programme of work to design and evaluate a menopausal education programme. This larger piece, required scientific evidence in a timely manner in order to support service delivery (Moons et al. 2021). A rapid review was identified as the most appropriate method to provide a resource efficient approach whilst maintaining quality in processes (Garritty et al. 2020). Therefore, the current rapid review aimed to (1) identify the structure and components of such programmes, and (2) summarise evidence of effectiveness of these programmes on menopausal knowledge, symptoms and quality of life for women.

## Methods

This study employed Cochrane methods for rapid reviews (Garritty et al. 2020) to facilitate the rapid development and dissemination of findings. Key stakeholders, including staff from voluntary sector organizations with experience in developing and delivering educational programmes, were involved in formulating the research question and establishing the eligibility criteria for the review.

### Data sources

Systematic searches were conducted in four databases (Cochrane Central, Medline, Embase, CINAHL) on 31 January 2023. The search strategy was developed with a subject librarian and included multiple search terms linked to pre, peri, and postmenopausal women and educational support programmes. Due to lack of resources for translation, all searches were limited to the English language. An example search strategy for Medline is detailed in Table 1. The search strategies for all electronic databases are provided in supplementary material 1.

**Table 1:** Medline search strategy

Set	Search statement
1	exp Climacteric/
2	(menopaus* OR postmenopaus* OR premenopaus* OR perimenopause* OR climacteric).mp
3	1 OR 2
4	(education* program* OR education* intervention* OR education* therap* OR education* treatment* OR education* tool*).mp
5	3 AND 4
6	limit 5 to English language

### Eligibility criteria

Studies were included if they (1) were primary research employing quantitative methods, (2) involved adults (age 18 +) or adult caregivers/spouses, (3) evaluated menopause education programmes, and (4) reported on educational or menopausal-related outcomes. Secondary research such as systematic reviews and meta-analyses were excluded. To ensure the inclusion of studies that were focused on menopause education, studies were excluded if less than 50% of the intervention consisted of menopausal education or if they only included signposting. Articles not available in the English language were excluded due to lack of resources for translation.

### Screening and selection

Results from searches were imported to, and managed in, Covidence systematic review software (Veritas Health Innovation 2023). The standardised title and abstract form in Covidence (i.e. form containing options for include, maybe, exclude) was piloted using the same 50 abstracts for the screening team (KMcC, CMcF). The screening team then dual screened more than 20% (*n* = 239/836, 29%) of titles and abstracts (Cohen’s Kappa = 0.76, indicating substantial agreement), while the remaining titles and abstracts were screened by one reviewer (KMcC), according to the above eligibility criteria. A second reviewer (CMcF) screened all excluded abstracts for consensus. To calibrate and test the full text screening form in Covidence (i.e. form containing options for include and exclude with reason), 10% of relevant full texts were piloted by the entire team (KMcC, CMcF, KP). One reviewer (KMcC) then screened the remaining full texts, and two additional reviewers (CMcF, KP) screened all excluded full texts for verification. Any discrepancies were discussed among all authors until a consensus was reached.

### Data extraction

The TIDIER framework (Hoffmann et al. 2014) informed development of the data extraction form in Covidence. This form was piloted by three reviewers (KMcC, CMcF, KP) using six full texts to ensure relevance of data to be extracted. Data extracted included contextual study information, participant demographics, programme components, and both educational and clinical outcomes. A single reviewer (KMcC) extracted data and a second reviewer (CMcF, KP) checked correctness and completeness of all extracted data.

### Data synthesis

Data was synthesized using a narrative approach driven by the objectives of the review. As this was a rapid review, risk of bias was not assessed.

## Results

### Search results

**Fig. 1:**
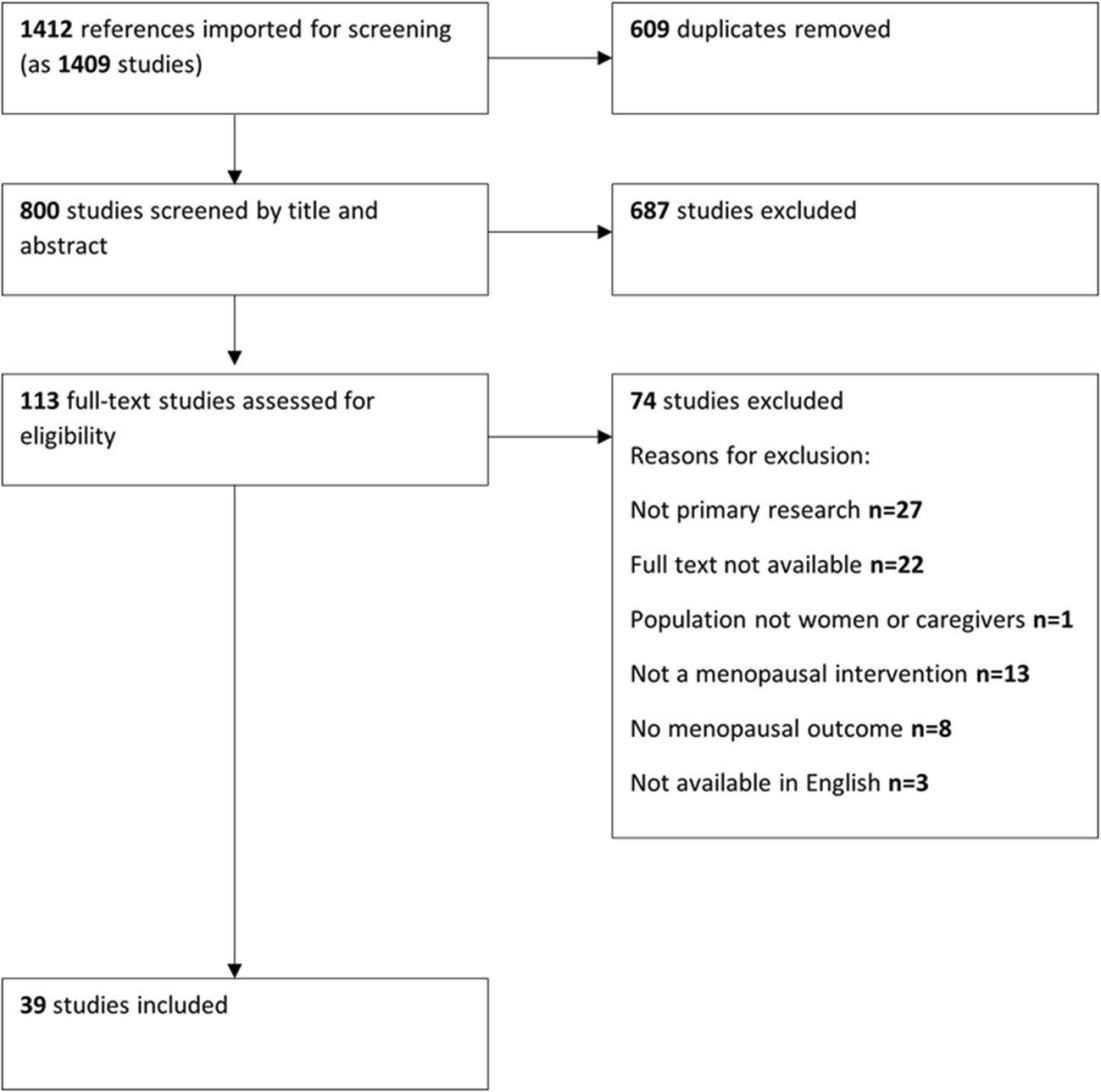
PRISMA diagram showing the flow of studies through the review

A total of 1,409 studies were retrieved from electronic database searches. After removing 609 duplicates, 800 studies underwent screening based on title and abstract. Subsequently, 113 studies underwent full text screening. Out of these, 74 studies were excluded, leaving 39 studies to be included in the review (Fig. 1).

### Characteristics of studies

Table 2 summarises the characteristics of studies included in this review. The included studies were published between 1995 and 2022. Two-thirds of the studies (*n* = 26/39, 66.7%) were published in the last 10 years. Almost half of the studies (*n* = 18/39, 46.2%) were conducted in Iran, with other locations including Africa (*n* = 1, 2.6%), Australia (*n* = 1, 2.6%), Bangladesh (*n* = 1, 2.6%), China (*n* = 1, 2.6%), India (*n* = 3, 7.7%), Israel (*n* = 1, 2.6%), Japan (*n* = 2, 5.1%), South America (*n* = 2, 5.1%), Sri Lanka (*n* = 1, 2.6%), Sweden (*n* = 1, 2.6%), Taiwan (*n* = 1, 2.6%), Turkey (*n* = 1, 2.6%), UK (*n* = 1, 2.6%) and the USA (*n* = 4, 10.3%). Approximately half of the studies were Randomized Controlled Trials (*n* = 19/39, 48.7%), while the remaining studies consisted of single-group designs (*n* = 14/39, 35.9%) and non-randomized group studies (*n* = 6/39, 15.4%).

**Table 2:** Study Characteristics

Author and Year	Geographical location	Study design	Participants	Menopausal stage of women	Total sample size
Afshari 2020	Iran	Cluster RCT	Women	Peri / Post	68
Anderson 2015	Australia	RCT	Women	Peri / Post	55
Bahri 2016	Iran	RCT	Women; Caregivers	Peri / Post	109
Bahri 2018	Iran	RCT	Women	Peri / Post	68
Barriga 2008	South America	Pre-post single group	Women	Peri / Post	105
Bhattacharya 2016	India	Pre-post single group	Women	Pre	300
Esposito et al 2012	South America	Pre-post single group	Women	Peri / Post	69
Fallahipour 2022	Iran	Non-randomised group	Women	Peri / Post	140
Forouhari 2010	Iran	RCT	Women	Pre / Peri / Post	62
Gebretatyos 2020	Africa	Pre-post single group	Women	Not reported	99
Hossein et al 2022	Iran	RCT	Women	Peri / Post	100
Javadivala 2020	Iran	RCT	Women	Peri / Post	190
Karimi 2022	Iran	RCT	Women	Peri / Post	70
Khandehroo 2022	Iran	RCT	Women	Peri / Post	100
Koyuncu 2018	Turkey	Pre-post single group	Women	Pre / Peri / Post	54
Lemaire 1995	USA	Pre-post single group	Women	Pre / Peri / Post	177
Liao 1998	UK	RCT	Women	Pre	86
Moshki 2018	Iran	RCT	Women	Peri / Post	91
Moshki 2022	Iran	Non-randomised group	Women	Peri / Post	110
Naeij 2019	Iran	RCT	Women	Peri / Post	52
Nazari 2016	Iran	Pre-post single group	Women	Peri / Post	200
Patil 2022	India	Pre-post single group	Women	Pre	50
Rathnayake 2020	Sri Lanka	Non-randomised group	Women	Peri / Post	80
Rindner 2017	Sweden	RCT	Women	Peri / Post	131
Rotem 2005	Israel	Non-randomised group	Women	Peri / Post	82
Rothert 1997	USA	3-group RCT	Women	Pre / Peri / Post	379
Sasanpour 2020	Iran	Pre-post single group	Women	Peri / Post	50
Senba 2010	Japan	Non-randomised group	Women	Peri / Post	52
Shobeiri 2017	Iran	RCT	Women	Peri / Post	100
Sunny 2019	India	Pre-post single group	Women	Pre / Peri / Post	394
Trudeau 2011	USA	Pre-post single group	Women	Peri / Post	35
Tsao 2007	Taiwan	Non-randomised group	Women	Peri / Post	353
Ueda 2009	Japan	Pre-post single group	Women	Peri / Post	39
Vakili 2019	Iran	RCT	Women	Peri / Post	108
Wong et al 2018	China	RCT	Women	Peri / Post	197
Yarelahi 2021	Iran	RCT	Women; Caregivers	Peri / Post	80
Yasmin 2012	Bangladesh	Pre-post single group	Women	Peri / Post	205
Yoshany 2021	Iran	RCT	Women	Peri / Post	80
Zeolla 2004	USA	Pre-post single group	Women	Pre / Peri / Post	31

*RCT* randomised controlled trial

### Participants

A total of 4,751 participants (range 31–394, mean 136) were included across the 39 studies (Table 2). The majority of the studies included women (*n* = 37/39, 94.9%), while only two studies included women and their spouse/partner (*n* = 2/39, 5.1%). Most studies included peri- and/or postmenopausal women (*n* = 29/39, 74.4%), with 15.4% including pre-, peri-, and postmenopausal women (*n* = 6/39), 7.7% including premenopausal women only (*n* = 3/39), and one study not reporting the menopausal stage of participants.

### Structure and components of menopausal education programmes (aim 1)

#### Intervention delivery

More than two-thirds of the studies did not report the total duration of the intervention (*n* = 15/39, 38.5%). Among those that did, the duration ranged from 1 to 38 weeks, with a mean duration of 7 weeks. The number of sessions within the intervention ranged from 1 to 10, with a mean of 4 sessions. Nine studies (23.1%) did not report the number of sessions. Among the 17 studies (43.6%) that reported the frequency of sessions, the majority were carried out weekly (*n* = 12/17, 70.1%). The duration of individual sessions ranged from 30 to 240 min, with a mean duration of 87 min per session.

In terms of Programme delivery, the majority of studies delivered sessions in groups (*n* = 24/39, 61.5%). Other types of session delivery included individual (*n* = 7/39, 17.9%), combined group and individual (*n* = 6/39, 15.4%), joint with spouse/partner (*n* = 1/39, 2.6%), and one study did not report how sessions were delivered (2.6%). Almost one-quarter of studies did not report who delivered the sessions (*n* = 9/39, 23.1%), and an additional 3 studies were self-delivered (7.7%). Sessions were most frequently delivered by one of the authors/researchers (*n* = 10/39, 25.6%), a multidisciplinary healthcare team (*n* = 7/39, 17.9%), or a nurse/midwife (*n* = 5/39, 12.8%). The remaining sessions were delivered by the author/researcher and peers (*n* = 2/39, 5.1%), health educators (*n* = 1/39, 2.6%), a pharmacist (*n* = 1/39, 2.6%), or trained staff (*n* = 1/39, 2.6%).

The majority of sessions were delivered in person (*n* = 34/39, 87.2%). Almost half of the studies employed group discussions (*n* = 17/39, 43.6%). More than two-thirds of the studies used a brochure/written format and/or presentations (*n* = 15/39, 38.5%). Other methods of delivering Programmes included question and answer sessions (*n* = 7/39, 17.9%), emails/text messages/letters (*n* = 6/39, 15.4%), telephone calls or audio-recorded information or physical activity (*n* = 5/39, 12.8%), videos/movies or journals/diaries/notebooks (*n* = 4/39, 10.3%), and role-playing (*n* = 2/39, 5.1%). Supplementary material 2 summarises the interventions from included studies.

#### Intervention topics

The most frequently covered topics within the Programmes included signs and symptoms of menopause (*n* = 25/39, 64.1%), treatment/management of menopause (*n* = 20/39, 51.3%), lifestyle factors (e.g., diet, physical activity), physiology of menopause (*n* = 15/39, 38.5%), risks and other conditions associated with menopause (*n* = 15/39, 38.5%), definition of menopause (*n* = 12/39, 30.8%), stress management (*n* = 12/39, 30.8%), behaviour change and confidence (*n* = 7/39, 17.9%), self-care (*n* = 6/39, 15.4%), and supporting women before/during menopause (*n* = 4/39, 10.3%). Other less frequently reported topics included the anatomy of the female and male reproductive systems, sexual function, sexual relationships, health literacy skills, mindfulness, self-management, getting support, screening in menopause, mental health, and communication with healthcare professionals and spouse/partner (Supplementary material 2).

### Effectiveness of menopausal education programmes (aim 2)

#### Outcomes measured

The most frequently reported outcomes included menopausal knowledge (*n* = 17/39, 43.6%), menopausal symptoms (*n* = 16/39, 41.0%), quality of life (*n* = 11/39, 28.2%), and psychological well-being, including anxiety, depression, self-efficacy, self-acceptance, self-esteem, and perceived stress (*n* = 9/39, 23.1%). Other outcomes included but were not limited to general health (*n* = 6/39, 15.4%), attitudes towards menopause (*n* = 5/39, 12.8%), menopausal uncertainty (*n* = 3/39, 7.7%), anthropometric measurements (*n* = 3/39, 7.7%), physical activity (*n* = 3/39, 7.7%), sexual function (*n* = 2/39, 5.1%), and self-care (*n* = 2/39, 5.1%). Supplementary material 3 summarises outcomes and results from included studies.

#### Menopausal knowledge

All 17 studies that measured menopausal knowledge demonstrated statistically significant improvements post-intervention. Most of these studies were single-group design studies (*n* = 9/17, 52.9%), with six Randomized Controlled Trials (*n* = 6/17, 35.3%) and two non-randomized group designs (*n* = 2/17, 11.8%). Many programmes were delivered by the researcher/author (*n* = 10/17, 58.8%) however the delivery methods and content varied across the studies (see Table 3).

**Table 3:** Overview of 17 studies that measured menopausal knowledge

Delivery structure	
Group	8
Individual	5
Combined	3
Not reported	1
Delivery method	
In-person	14
Online	3
Education methods	
Brochure	6
Presentations	6
Group discussions	6
Videos/ movies	4
Questions and answers	4
Content	
Signs and symptoms	11
Treatment and management	10
Risks/conditions associated with menopause	7
Lifestyle factors e.g. diet/ physical activity	5

The studies that demonstrated significantly improved menopausal knowledge included group (*n* = 8/17, 47.1%), individual (*n* = 5/17, 29.4%), and combined group and individual (*n* = 3/17, 17.6%) programmes (one study did not report the method of delivery). They were delivered in various ways, including in person (*n* = 14/17, 82.4%), supplemented by brochure/written information (*n* = 6/17, 35.3%), presentations (*n* = 6/17, 35.3%), group discussions (*n* = 6/17, 35.3%), videos/movies (*n* = 4/17, 23.5%), and question and answer opportunities (*n* = 4/17, 23.5%). Many programmes were delivered by the researcher/author (*n* = 10/17, 58.8%).

The most frequently reported topics in the programmes were signs and symptoms of menopause (*n* = 11/17, 64.7%), treatment and management (*n* = 10/17, 58.8%), risks and other conditions associated with menopause (*n* = 7/17, 41.2%), definition and physiology of menopause (*n* = 6/17, 35.3%), and lifestyle factors such as diet and physical activity (*n* = 5/17, 29.4%).

#### Menopausal symptoms

The majority of studies (*n* = 13/16, 81.3%), reported a statistically significant reduction in menopausal symptoms post-intervention. There is heterogeneity in reporting across symptoms and therefore it is not possible to report specific symptoms addressed. While the results of the remaining studies did not reach statistical significance, they did indicate trends toward symptom improvement. It's worth noting that all these studies were conducted in person by healthcare professionals. While the results of the remaining studies did not reach statistical significance, they did indicate trends toward symptom improvement. It's worth noting that all these studies were conducted in person by healthcare professionals.

#### Quality of life

All studies measuring quality of life, including mental health, reported statistically significant improvements after the education intervention. All interventions were delivered in person, except for one that was delivered via CD/audio-recording. Additionally, more than half of the interventions included information in a brochure/written format in addition to in-person sessions (*n* = 6/11, 54.5%).

## Discussion

This rapid review identified 39 primary research studies evaluating menopause education programmes on menopausal knowledge, symptoms and quality of life in women. Most studies included within this review were published in the last 10 years, highlighting the novelty of research activity in this area. The rationale for including a broad date range of papers stems from their shared measurement of fundamental concepts such as physical and psychological symptoms, albeit with variations in specific symptoms; for instance, while some studies focus on hot flushes, others emphasise pain. This also highlights the challenge posed by poor reporting practices, which may obscure potential shifts in the content and effectiveness of education programmes over time.

While nearly half of the studies (46.2%) were conducted in Iran, it's essential to acknowledge that these findings can serve as a basis for understanding comparable situations in other regions. The socio-economic, cultural, and geopolitical factors impacting Iran are not entirely unique, therefore the results obtained from these studies can offer valuable insights applicable to other countries facing similar challenges or sharing similar characteristics. An improvement in knowledge, menopausal symptoms and quality of life outcomes were demonstrated across most studies.

Intervention parameters and components varied, yet their overall reporting lacked detail, omitting crucial information such as the identity of the intervention deliverer and their credentials. This deficiency impedes replication and implementation efforts. Adhering to guidelines like TIDieR would enhance transparency in the research process, especially concerning the assessment of intervention delivery bias (Garritty et al. 2020). Additionally, details regarding intervention delivery, such as session duration and frequency, were inadequately documented. Despite identifying different methods and topics for inclusion in menopausal education programmes, this review highlighted the lack of consistent and comprehensive reporting of the topics within each programme. These findings highlight the need to enhance intervention reporting in future studies. Following guidelines such as TIDieR could improve clarity and reproducibility in intervention reporting, thereby elevating the quality of research within this domain (Garritty et al. 2020).

Whilst intervention delivery and components varied across the evidence base, group education emerged as a prominent method of delivering menopause education in the reviewed studies. While group education was a relatively common method of delivering menopause education, it is not possible to draw conclusions on effectiveness due to lack of quality appraisal in this rapid review. Group education may, however provide several benefits over 1–1. Research suggests that group sessions provide several advantages, such as creating a supportive environment, enabling peer interactions, and fostering shared experiences among participants (Aninye et al. 2021; Ayers et al. 2010; Wilberforce et al. 2018). The group dynamic can contribute to increased engagement, motivation, and overall satisfaction with the educational programme (Ayers et al. 2010). Group education has also been outlined to offer cost-effectiveness and scalability advantages, allowing the dissemination of information to a larger, more diverse number of women simultaneously (Barron et al. 2019), thus making it a practical approach in community and healthcare settings. The sharing of resources, experiences, and coping strategies among groups also contribute to self-management and long-term support (Bélanger et al. 2011). Programmes delivered by healthcare professionals, in person, and incorporating interactive components were also common, suggesting use of a range of educational strategies and participant engagement are useful approaches for improved outcomes (Golyan Tehrani et al. 2007; Li et al. 2023; Liao and Hunter 1998; Keye et al. 2023; Shobeiri et al. 2017; Yazdkhasti et al. 2015).

Research consistently emphasises the significance of educating women about the signs and symptoms of menopause (Gebretatyos et al. 2020; Li et al. 2023; Keye et al. 2023; Shobeiri et al. 2017; Tariq et al. 2023), as well as providing information on treatment options, thus empowering them to take control of their healthcare (Tariq et al. 2023). These were common topics included within the reviewed studies in addition to lifestyle factors, such as diet and physical activity, being recognised as important aspects of managing menopause symptoms and improving overall well-being including mental health (World Health Organization 2022; Ye et al. 2022). Addressing the risks and associated conditions of menopause is also crucial for promoting women's health during this stage of life (National Institute for Health and Care Excellence 2015) with topics such as sexual function, mental health, and communication with healthcare professionals and partners/spouses, important considerations in menopause education (Keye et al. 2023; Shobeiri et al. 2017). However, these topics were less commonly covered by studies included in this review. Given the impact and prevalence of menopausal symptoms, which directly affect sexual function and mental health (Woods and Utian 2018; Ye et al. 2022), this warrants consideration in the development of future menopausal education programmes.

Nonetheless, findings from this review suggest menopausal education programmes can increase women’s knowledge of menopause concurrently with a reduction in the severity of symptoms. This finding is consistent with previous research that highlights the negative relationship between level of knowledge and symptom severity (Tariq et al. 2023; Afshari et al. 2020), thus highlighting the need for menopausal education programmes to enhance symptom management for women. In addition, menopausal education programmes can improve quality of life for women (Keye et al. 2023). Despite these positive findings, the most recent NICE guidelines (National Institute for Health and Care Excellence 2015) recommend the provision of information and advice but fail to include specific educational programmes as a recommendation for clinical practice. Subsequent revisions to these guidelines should include menopausal education interventions to improve quality of life for women before, during and after menopause.

Most studies focused on delivering interventions to peri- and/or postmenopausal women, despite broader research indicating the importance of early intervention in health education (Aninye et al. 2021). This observation likely stems from the more pronounced symptoms experienced by women in these stages, prompting them to seek information and support. Healthcare providers may prioritise education and interventions for women currently experiencing menopause, but research recognises the importance of educating premenopausal women as well, to prepare them for the transition and potentially mitigate future challenges (Afshari et al. 2020; Moore et al. 2023). The findings of this review therefore highlight a gap in the evidence in relation to clearly defined intervention parameters tailored to premenopausal women. Equipping them with knowledge about menopause can empower them to manage symptoms effectively (Keye et al. 2023). Additionally, by scrutinising the methodologies and outcomes of these studies, researchers can identify principles and strategies that are transferable across diverse settings, thereby enhancing the relevance and applicability of the findings beyond Iran.

In addition, health education programmes should be inclusive and holistic (Moore et al. 2023) yet included studies did not report inclusion of women with complex and/or additional needs who may have challenges or requirements, such as communication, cognitive, or behavioural difficulties, beyond those typically experienced during menopause (Moore et al. 2023). These women will still experience menopause and thus future interventions should be inclusive and accessible for all regardless of healthcare needs. Clinical guidelines recommend providing information to women themselves and their family members or carers/caregivers, including children and adults, who care for a family member, partner, or friend due to illness, frailty, disability, mental health problems, or addiction and cannot cope without support (National Institute for Health and Care Excellence 2015; NHS England n.d.). However, only two studies in this review incorporated carers/spouses into the education programme. Future educational programmes should consider the inclusion of carers/spouses to maximise outcomes for women, and this should be especially considered when including women with complex and/or additional needs.

## Limitations

This rapid review has certain limitations. Almost half of the studies (*n* = 18/39, 46.2%) were conducted in Iran. This makes the findings very specific as they largely apply to one country. The search was limited to English-language publications, potentially excluding relevant studies published in other languages. As per Cochrane guidelines for Rapid Reviews (Garritty et al. 2020), authors were not contacted for studies not available in English and therefore may be considered as a limitation of this review. The focus on rapid review methodology may have limited the depth of data extraction and analysis compared to a full systematic review however the methodology followed the Cochrane guidelines for Rapid Reviews (Garritty et al. 2020), resulting in the quality of this review remaining high. Quality appraisal was not conducted, which can be identified as a limitation with a rapid review approach. The heterogeneity among the included studies was unsurprising and posed challenges in synthesising the findings and thus recommendations for future research and practice should be interpreted with caution. Whilst the number of studies included in the review provides a platform on which to base conclusions, quality appraisal was not conducted, and in conjunction with poor reporting of intervention parameters, limits the reliability of conclusions.

## Conclusion

In conclusion, this rapid review identified a diverse range of menopause education programmes addressing topics such as signs and symptoms, treatment and management, lifestyle factors, and the physiology of menopause. Most studies included peri- and postmenopausal participants, with group education being a prominent delivery method. The included studies support the use of education programmes for improving menopausal knowledge, symptoms, and quality of life post-intervention. However, the lack of comprehensive reporting of intervention components hinders the replication and implementation of programmes. Moving forward, it is crucial to include premenopausal women in education programmes, adhere to reporting guidelines, and ensure interventions are inclusion and accessible for all. This will contribute to the development of evidence-based and replicable menopause education programmes that may effectively support women's understanding and management of menopause.

## Supplementary information

Below is the link to the electronic supplementary material.

Supplementary file1 (DOCX 23 KB)

Supplementary file2 (DOCX 36 KB)

Supplementary file3 (DOCX 35 KB)
