# Supplementary material for: A rapid review of menopausal education programmes
Source: Arch Womens Ment Health. 2024 May 25;27(6):975–83. doi: 10.1007/s00737-024-01476-8 (PMC11579047; doi:10.1007/s00737-024-01476-8)
Supplement: Supplementary file 1 — Supplementary file1 (DOCX 23 KB) [file 737_2024_1476_MOESM1_ESM.docx]

Supplementary Information 1: The search strategies for all electronic databases

Medline search strategy

| Set | Search statement |
| --- | --- |
| 1 | exp Climacteric/ |
| 2 | (menopaus* OR postmenopaus* OR premenopaus* OR perimenopause* OR climacteric).mp |
| 3 | 1 OR 2 |
| 4 | (education* program* OR education* intervention* OR education* therap* OR education* treatment* OR education* tool*).mp |
| 5 | 3 AND 4 |
| 6 | limit 5 to English language |

Embase search strategy

| Set | Search statement |
| --- | --- |
| 1 | exp Climacterium/ |
| 2 | (menopaus* OR postmenopaus* OR premenopaus* OR perimenopause* OR climacteric).mp |
| 3 | 1 OR 2 |
| 4 | (education* program* OR education* intervention* OR education* therap* OR education* treatment* OR education* tool*).mp |
| 5 | 3 AND 4 |
| 6 | limit 5 to English language |

CINAHL search strategy

| Set | Search statement |
| --- | --- |
| 1 | MH Climacteric+ |
| 2 | TX (menopaus* OR postmenopaus* OR premenopaus* OR perimenopause* OR climacteric) |
| 3 | S1 OR S2 |
| 4 | TX (education* program* OR education* intervention* OR education* therap* OR education* treatment* OR education* tool*). |
| 5 | S3 AND S4 |
| 6 | S3 AND S4 Narrow by language: - English |

COCHRANE database search strategy

| Set | Search statement |
| --- | --- |
| 1 | Title abstract keyword: climacteric |
| 2 | Or Title abstract keyword: menopaus* OR postmenopaus* OR premenopaus* OR perimenopause* OR climacteric |
| 3 | AND education* program* OR education* intervention* OR education* therap* OR education* treatment* OR education* tool*). |
