# Supplementary material for: A rapid review of menopausal education programmes
Source: Arch Womens Ment Health. 2024 May 25;27(6):975–83. doi: 10.1007/s00737-024-01476-8 (PMC11579047; doi:10.1007/s00737-024-01476-8)
Supplement: Supplementary file 2 — Supplementary file2 (DOCX 36 KB) [file 737_2024_1476_MOESM2_ESM.docx]

Supplementary Material 1: Summary of interventions from included studies

| **Author & Year** | **Duration (weeks)** | **Total sessions** | **Session frequency** | **Session duration (minutes)** | **Mode of delivery** | **Delivery methods** | **Delivered by** | **Topics included** |
| --- | --- | --- | --- | --- | --- | --- | --- | --- |
| Afshari 2020 | 2 | NR | NR | NR | Individual | CD/audio-recorded; Videos / movies; Telephone calls; Emails / messages / letters; Self-directed | N/A | Physiology of menopause; Signs and symptoms of menopause; Treatments; Self-care |
| Anderson 2015 | 12 | 3 | Every 6 weeks | NR | Individual | Face to face; Brochure/written; Journal / diary / notebook; Telephone calls; Emails / messages / letters; Website | Nurse | Signs and symptoms of menopause; Treatments; Lifestyle; Behaviour change & confidence |
| Bahri 2016 | NR | 3 | NR | 60 | Group | Face to face; Brochure/written; Videos / movies; Presentation | Researcher / author | Signs and symptoms of menopause; Treatments; Risks and diseases associated with menopause; Supporting women before / during menopause |
| Bahri 2018 | NR | 4 | NR | 45 | Group | Face to face | Researcher / author | Risks and diseases associated with menopause; Self-care; Other |
| Barriga 2008 | NR | NR | NR | NR | NR | Face to face; Presentation | NR | Signs and symptoms of menopause; Lifestyle; Risks and diseases associated with menopause |
| Bhattacharya 2016 | 1 | NR | NR | NR | Individual | Self-directed | N/A | Definition of menopause; Signs and symptoms of menopause; Treatments |
| Esposito et al 2012 | 38 | 7 | Every 45 days | 120 | Individual; Group | Face to face; Presentation; Group discussion | Psychologist; Reproductive health professional; therapist, nutritionist | Physiology of menopause; Signs and symptoms of menopause; Treatments; Lifestyle; Stress management; Risks and diseases associated with menopause |
| Fallahipour 2022 | NR | 5 | NR | 60 | Group | Brochure/written; Presentation; Group discussion; practical physical activities (aerobic, stretching, walking) | Trained staff | Signs and symptoms of menopause; Lifestyle; Behaviour change & confidence; environmental evaluation, self-evaluation, self-efficacy (under behaviour confidence) |
| Forouhari 2010 | 6 | 6 | Weekly | 45-60 | Group | Face to face; Brochure/written; CD/audio-recorded | NR | Definition of menopause; Physiology of menopause; Signs and symptoms of menopause; Treatments; Lifestyle; Stress management; Risks and diseases associated with menopause; Information about female genital organs |
| Gebretatyos 2020 | 4 | 3 | NR | 60 | Group | Face to face; Presentation; Group discussion | Midwife | Definition of menopause; Signs and symptoms of menopause; Lifestyle |
| Hossein et al 2022 | 4 | 4 | Weekly | 90 | Group | Face to face; Telephone calls; Presentation; Group discussion | NR | Self-care |
| Javadivala 2020 | 12 | 3-8 | NR | NR | Group | Face to face; Group discussion; Physical activity program | NR | Signs and symptoms of menopause; Treatments; Lifestyle; Risks and diseases associated with menopause; Behaviour change & confidence |
| Karimi 2022 | 1 | 5 | NR | 45-120 | Group | Face to face; Brochure/written; Telephone calls; Emails / messages / letters | NR | Definition of menopause; Physiology of menopause; Signs and symptoms of menopause; Lifestyle; Stress management; Risks and diseases associated with menopause; Self-care; marital relationships, communicating with spouse |
| Khandehroo 2022 | 4 | 4 | Weekly | 240 | Group | Face to face; Roleplaying; Group discussion; teamwork | Health educators | Treatments; Health literacy skills |
| Koyuncu 2018 | NR | 3 | NR | 30 | Group | Face to face; Videos / movies; demonstration, Q&A | Researcher / author | Definition of menopause; Risks and diseases associated with menopause; Not specifically stated |
| Lemaire 1995 | NR | NR | NR | NR | Group | Face to face; Presentation; Q&A | Nurse; Reproductive health professional; endocrinologist, general surgeon | Physiology of menopause; Treatments; Risks and diseases associated with menopause |
| Liao 1998 | NR | 2 | NR | 90 | Individual; Group | Face to face; Brochure/written; CD/audio-recorded; Group discussion; Q&A | Researcher / author | Treatments; Lifestyle; Stress management; Risks and diseases associated with menopause; Behaviour change & confidence; menopause transition, knowledge, beliefs and expectations about menopause and ageing |
| Moshki 2018 | 4 | 4 | Weekly | 120 | Group | Face to face; Brochure/written; Emails / messages / letters | Researcher / author | Signs and symptoms of menopause; Necessary care to be taken during this period as well as instructions on how to be happy and have positive social relations during the menopause period |
| Moshki 2022 |  |  |  |  | Individual; Group | Face to face; Brochure/written; CD/audio-recorded; Presentation; Group discussion; Q&A | NR | Not specifically stated |
| Naeij 2019 | 5.5 | 4 | Every 10 days | 70 | couples | Face to face; counselling education program, couple assignments/tasks | Midwife | Definition of menopause; Physiology of menopause; Signs and symptoms of menopause; sexual function and dysfunction, factors affecting sexual function in menopause, positive and negative stimuli in healthy and unhealthy sexual function, myths about sex in menopause |
| Nazari 2016 | NR | 4 | NR | NR | Group | Face to face; Brochure/written; Presentation; Group discussion | NR | Lifestyle; Stress management; interpersonal relationships, spiritual growth and responsibility health |
| Patil 2022 | NR | NR | NR | NR | Individual | Face to face | Researcher / author | Not specifically stated |
| Rathnayake 2020 | 8 |  |  | 60 | Individual; Group | Face to face; Journal / diary / notebook; Presentation; Group discussion; visual images | Researcher / author | Signs and symptoms of menopause; Treatments; Lifestyle; Stress management |
| Rindner 2017 | 2 | 2 | Weekly | 120 | Group | Face to face; Group discussion; Q&A | Nurse; DistrictMidwife | Definition of menopause; Physiology of menopause; Signs and symptoms of menopause; Risks and diseases associated with menopause; Self-care; hormonal transition, mental unhealth, sleep, relationship, sexual health and desire |
| Rotem 2005 | 10 | 10 | Weekly | 120 | Group | Face to face; Presentation; Roleplaying; Group discussion; Group tasks | Medical and paramedical professionals | Physiology of menopause; Treatments; Lifestyle; Stress management; sociocultural perspective, elder care and resulting changes within the nuclear family, couplehood, intimacy and sexuality |
| Rothert 1997 | 3 | 3 | Weekly | 90 | Individual; Group | Face to face; Brochure/written; Presentation; Group discussion; workbook, Q&A, active involvement and Individual activities | Researcher / author | Definition of menopause; Physiology of menopause; Signs and symptoms of menopause; Treatments; Risks and diseases associated with menopause; Self-care; Behaviour change & confidence; Communication with healthcare professionals |
| Sasanpour 2020 | NR | 6 | Weekly | 60-90 | Group | Face to face; Q&A | Midwife | Physiology of menopause; Signs and symptoms of menopause; Treatments; Risks and diseases associated with menopause; Sexual intercourse during menopause, sexual dysfunction |
| Senba 2010 | 26 | 6 | Monthly | 120 | Group | Face to face; Journal / diary / notebook; Group discussion; interactive participation | Doctor and Midwife | Physiology of menopause; Signs and symptoms of menopause; Stress management; Need for healthcare during menopause, action plan for future health care |
| Shobeiri 2017 | 3 | 5 | NR | 45-60 | Group | Face to face; Brochure/written; Presentation; educational photos | NR | Definition of menopause; Signs and symptoms of menopause; Treatments; Lifestyle; Screening in menopause - pap smear, mammography, Education to their spouse and the best of friend about menopausal symptom and action plan for health care in menopause |
| Sunny 2019 | 2 | 2 | After two weeks | 60 | Group | Face to face; home practice log sheet, yoga, relaxation and breathing techniques | Researcher / author | Lifestyle; Stress management; Yoga |
| Trudeau 2011 | NR | NR | NR | NR | Individual | Online (synchronous); Self-directed | N/A | Physiology of menopause; Signs and symptoms of menopause; Treatments; Lifestyle; get support (list of resources, where women can offer solutions/suggestions, online tutorial about menopause), self-management, provider communication |
| Tsao 2007 | NR | 3+ | NR | 30-60 | Individual | Face to face; Brochure/written; Telephone calls | Researcher / author | Physiology of menopause; Treatments; Risks and diseases associated with menopause; Self-care; understanding menopause, perspectives of perimenopause |
| Ueda 2009 | 6 | 6 | Weekly | 90 | Group | Face to face; Presentation; practical / exercise sessions | Nurse; Reproductive health professional; medical technologist, physician, dietician, exercise teacher | Physiology of menopause; Signs and symptoms of menopause; Lifestyle; Behaviour change & confidence; Exercise, measuring BP and HR, interpreting lab data |
| Vakili 2019 | 2 | 2 | Weekly | 120 | Group | Face to face | Researcher / author; peers | Anatomy of the female and male reproductive system, sexual function, sexual relationship, effect of menopause on sexual function, confronting and coping with sexual dysfunction |
| Wong et al 2018 | 8 | 8 | Weekly | 150 | Individual; Group | CD/audio-recorded; Home exercises | Nurse; Psychologist | Stress management; Mindfulness: body scan, sitting meditation, mindful stretching exercises. |
| Yarelahi 2021 | NR | 4 | NR | 45 | Group | Face to face; Brochure/written; Emails / messages / letters; Group discussion; active participation of couples | NR | Definition of menopause; Physiology of menopause; Signs and symptoms of menopause; Stress management; Supporting women before / during menopause; sexual problems |
| Yasmin 2012 | NR | NR | NR | NR | Group | Face to face; Videos / movies; Group discussion | Researcher / author; Peer | Definition of menopause; Signs and symptoms of menopause; Treatments |
| Yoshany 2021 | 5 | 5 | Weekly | 45 | Group | Face to face; Brochure/written; Journal / diary / notebook; Emails / messages / letters; Presentation; Group discussion | Psychologist; Reproductive health professional | Signs and symptoms of menopause; Treatments; Lifestyle; Supporting women before / during menopause; Behaviour change & confidence |
| Zeolla 2004 | 1 | 1 | NR | 30 | Individual | Face to face; Brochure/written | Pharmacist | Definition of menopause; Signs and symptoms of menopause; Treatments; Risks and diseases associated with menopause |

NR: not reported, N/A: not applicable
