# Supplementary material for: A rapid review of menopausal education programmes
Source: Arch Womens Ment Health. 2024 May 25;27(6):975–83. doi: 10.1007/s00737-024-01476-8 (PMC11579047; doi:10.1007/s00737-024-01476-8)
Supplement: Supplementary file 3 — Supplementary file3 (DOCX 35 KB) [file 737_2024_1476_MOESM3_ESM.docx]

Supplementary material 2: Outcomes and results from included studies

| **Author & Year** | **Outcomes measured** | | | | | **Results** |
| --- | --- | --- | --- | --- | --- | --- |
|  | **Knowledge** | **Symptoms** | **Quality of life** | **Psychological outcomes** | **Other outcomes** |  |
| Fallahipour 2022 |  | X |  | X | X | Significant decreases in physical symptoms, sleep problems, muscle problems, discomfort, psychological symptoms, depression, irritability, and anxiety in the intervention group postintervention. |
| Afshari 2020 | X |  |  |  | X | Significant increase in knowledge about menopause over time but no significant difference between the two groups re knowledge immediately after and one month after intervention. Decrease in uncertainty immediately after intervention in both groups, but the score increased one month after intervention, and these changes were statistically significant. No significant difference between the two groups in terms of uncertainty about menopause immediately and one month after intervention. |
| Sasanpour 2020 |  |  |  |  | X | The mean score of sexual dysfunction beliefs in postmenopausal women after education was significantly increased. |
| Vakili 2019 |  |  |  |  | X | Mean total score of sexual function and its domains was significantly increased in the group and peer education groups after the intervention compared with before the intervention. However, there was no significant difference 1 month after in control group. |
| Moshki 2022 |  |  |  | X |  | Average attitude score in the three groups was significantly different after intervention. Mean of self-efficacy score was significantly different in the three groups after intervention. Psychological wellbeing increased after the intervention and this increase was also statistically significant. |
| Bahri 2018 |  |  |  |  | X | Significant increase in the score of knowledge in the intervention group compared to that in the controls 1 month after the intervention. The change in the score of the knowledge of self-care during menopause between baseline and 1-month intervention was significant between the two groups. The scores of self-care activities significantly increased in the intervention group compared to those in the controls one month after the intervention. The change in the score of self-care activities before and after the intervention was significant between the two groups. |
| Javadivala 2020 |  | X |  | X |  | Intervention group showed a significant reduction in the frequency and severity of all menopausal symptoms rated severe or very severe. In the non-intervention group, hot flushes, sleep problems and joint problems were significantly worse. Intervention programme significantly improved the physical activity self-efficacy score, while no significant difference was found in the non-intervention group. |
| Hossein et al 2022 |  |  | X |  | X | Post-test mean scores of QOL and all its subscales in the intervention group were significantly greater than the control group. Post-test mean scores of self-care and all its subscales in the intervention group were significantly higher than the control group. |
| Karimi 2022 |  | X | X |  |  | Postintervention significant differences between groups in both of severity menopause symptoms and marital satisfaction parameters. Post intervention significant differences in severity of symptoms and marital satisfaction in intervention group but not control group. |
| Yarelahi 2021 | X |  | X | X |  | Menopausal knowledge of men (caregivers) in the intervention group increased significantly. Significant increase in the menopausal knowledge score of women in intervention and control groups. Increase in the knowledge score in the intervention group was significantly higher than the control group. After two months, the mean scores of dyadic coping in the women in the intervention group significantly increased compared to the control group. After the intervention, the mean scores of QoL in all subscales, and the total score in the intervention group were significantly lower than the control group. After the intervention, the mean score of QOL in all subscales and the total score in the control group did not change significantly although a significant decrease was observed in the intervention group. |
| Yoshany 2021 |  |  | X |  |  | Significant difference was found between the two groups immediately and 3 months post intervention. In the intervention group, a significant difference was found in QoL between before intervention and immediately after the intervention, while the before and 3 months after the intervention showed no significant differences. There was even a more significant difference between the immediately after the intervention and 3 months after the intervention. In the control group, there was a significant difference between QOL before and those immediately after intervention, as well as the scores before and those 3 months after the intervention. No significant difference was found between the scores immediately after the intervention and those after 3 months. The mean score of vasomotor, psychosocial, physical, and sexual domains of the women before the intervention was not significantly different in the intervention and control groups; however, after the intervention, a statistically significant difference was observed between the intervention and control groups regarding the mean score of all QOL domains. |
| Gebretatyos 2020 | X |  |  |  | X | Knowledge scores were significantly higher at immediately post-intervention, at 3-months follow-up and immediately post-intervention. The mean score of attitude had increased at immediately post-intervention and 3 months follow up. |
| Rathnayake 2020 | X |  |  |  | X | Knowledge and attitude scores increased in the intervention group during the intervention. In the control group, marginal increase in all dimensions of knowledge scores was seen, scores related to attitude remained unchanged. All MENQOL scores decreased during the follow-up in the intervention group except the sexual domain. Except the social functioning and comfort domains, all other domains of QOL and the overall QOL scores increased in the intervention group during the study period. Between-group comparison at the end of the 6-monthfollow-up showed an improvement of knowledge, attitude, MENQOL except sexual domain, and the overall QOL in the intervention group compared to the control group. |
| Naeij 2019 |  |  |  |  | X | Significant difference in sexual function between the intervention and control groups at follow up. A significant improvement was also observed in all the FSFI domains in the intervention group compared with the control group. |
| Koyuncu 2018 | X | X |  |  |  | Decrease in the somatic and psychological subscales of menopause rating scale and total score according to the total pre-education scores. No change in the urogenital subdimension. Positive changes were observed in the levels of knowledge about menopause and two sub-scales of the menopause attitude assessment scale. Significant increase in knowledge after intervention. |
| Moshki 2018 | X |  |  | X |  | Mean of knowledge score in the two groups before and after indicated significant effect of intervention on the level of knowledge of the participants in the test group. Significant differences in the mean scores of predisposing, reinforcing, and enabling factors as well as self-efficacy and self-acceptance before and after the intervention. |
| Rindner 2017 |  | X |  | X |  | The intervention group experienced a light reduction in symptoms while the control group mostly experienced the opposite. However, the difference between groups in changed symptoms did not reach statistical significance when calculating data as intention to treat nor when calculating as per protocol. |
| Shobeiri 2017 |  |  | X |  |  | There was not a significant difference in the QOL mean scores in before of the intervention between the two groups of intervention and control in all dimensions of QOL. There was a significant difference in the QOL mean scores between the two groups in immediately after the intervention and 3 months after the intervention in dimension of vasomotor, psychosocial, sexual and physical. |
| Bahri 2016 | X | X | X |  |  | Postintervention men's (caregiver's) knowledge scores were significantly higher in the intervention group. Significant improvement in husband's performance about menopause in intervention group post 3 months. In the intervention group, significant differences in the spouses knowledge and performance scores before and 3 months after. No such significant differences were detected in the control group. At 3 months post intervention, the mean score of the MENQOL questionnaire was significantly lower in intervention group than in the control group. |
| Anderson 2015 |  | X | X |  |  | Women in the intervention group reported a moderate improvement in somatic symptoms, vasomotor symptoms, and sexual dysfunction across time compared with controls. Women in intervention group reported moderate improvements in HR-QoL and sleep. All participants reported increased physical activity although a greater proportion of women in the intervention group reported increasing their activity levels compared with the control group - not statistically significant. |
| Bhattacharya 2016 | X |  |  |  |  | Significant increase in menopausal knowledge. |
| Nazari 2016 |  | X |  |  | X | No significant difference between the two groups regarding the mean scores of health-promoting lifestyles and mean scores of menopause rating scale (MRS) before the intervention but a significant difference was observed in this respect after the intervention. A significant difference in the intervention group's mean scores of health-promoting lifestyles and mean scores of MRS before and after the intervention but no significant difference was found in this regard in the control group. |
| Esposito et al 2012 |  | X |  |  | X | In the final period, there was a significant difference in the late postmenopausal group for vasomotor and nervousness symptoms. The total score in the Kupperman menopausal index was reduced significantly in both groups throughout the study. The women in both groups maintained similar scores at the initial and final periods, thus demonstrating commitment to quality of life in both cases. The domains most perceived by the patients in the two groups were depressive mood, attractiveness, and menstrual symptoms. There was a significant reduction in the depressive mood domain in both groups at the final period. There was no predominance of menopausal symptom intensity in the women's health questionnaire domains between the identified health factors in the two groups and the different evaluation times. |
| Forouhari 2010 |  | X | X |  |  | After educational intervention, a salient improvement was seen in the mean score for vasomotor symptoms compared to the scores prior to intervention. In the control group, comparing the scores before study, after study showed a statistically significant drop. The score for psychosocial function in the study group improved after intervention. However, in the control group a significant deterioration was registered. Three months after intervention, mean scores for physical wellbeing compared to base scores in the study group showed a significant improvement. In the control group, it showed a significant negative growth. In terms of sexual health, the study group showed a significant improvement. Whereas control group showed a significant opposite movement. Three months after education, score for QOL in the study group was improved significantly. Nevertheless, control group showed a significant deterioration in their sense of wellbeing. There was a statistically significant difference between study and control groups, three months after educational intervention, according to the mean score for vasomotor symptoms, psychosocial aspect, physical wellbeing, sexual health, and QOL. |
| Trudeau 2011 | X |  |  |  |  | Significant improvement knowledge about menopause following use of the Chart the Change demonstration program. |
| Senba 2010 |  | X |  |  |  | Simplified menopausal index (SMI) scores, physical symptoms of menopause and psychological symptoms all significantly improved from pre-test value to post-test and follow-up values. While the post-test Hospital Anxiety and Depression Scale (total) tended to improve in the intervention group, the post-test values of the control group tended to worsen. Post-test general health perceptions and follow-up role physical/role emotional significantly improved in both groups. However, post-test bodily pain and follow-up physical functioning, role physical, bodily pain and general health perceptions significantly improved only in the intervention group. |
| Ueda 2009 |  | X | X |  | X | The mean Simplified Menopausal Index (SMI) scores showed no significant changes before and after, and 1 year after the programme. Significant differences were observed in the subscale score for general health perception and the summary score for the physical component summary, both of which increased over time. |
| Barriga 2008 | X |  |  |  |  | Significant increase in menopausal knowledge. |
| Tsao 2007 | X | X |  |  | X | Significant improvement in the post-test on health knowledge about perimenopause in intervention and control. Perceived uncertainty about perimenopause was lower but not significant in the post-test in both groups of women. The beneficial effects on frequency of practicing healthy behaviours were significant for women in the intervention group and all its subgroups, but not control. A significant decrease in perceived perimenopausal disturbances. On the practice of health behaviour, the intervention effect estimates revealed significant changes after a 3-month and 1-year follow-up. In terms of perceived uncertainty, the estimates of intervention effect showed significant changes after 1-year follow-up. |
| Rotem 2005 |  | X |  |  |  | Significant increase in attitudes from baseline to follow-up. Significant decrease in perceived severity of physiological, psychological, and social symptoms in intervention group. |
| Zeolla 2004 |  |  |  |  | X | Significant increase all components of the Management of Menopause survey, including the composite score. At 1 year, statistically significant increases were seen for all components except exposure to counselling. |
| Liao 1998 | X |  |  | X | X | Following the intervention, scores on depressed mood and to a lesser extent anxiety, for the prepared group, reduced to a level similar to the control groups. Mean knowledge scores differed significantly between intervention and control 1, and between intervention and control 2. For the intervention group, knowledge scores increased significantly between time 1 to time 2 and between time 1 and time 3. Scores on 4/5 of the negative belief about menopause items improved (became less negative) for the intervention group at the post-intervention assessments. Within group differences were significant but between group comparisons did not reach statistical significance. At time 2, however, the intervention group were less likely to express the belief that most women at menopause experience serious vasomotor and somatic symptoms. They were also more likely to mention positive or neutral changes such as cessation of menstrual periods. following the intervention, the prepared groups stereotyped beliefs about the impact of menopause were significantly modified. 5-year follow-up: significant differences in knowledge of menopause between prepared and control groups with prepared group having significantly greater knowledge. No significant differences between prepared and control women in health and health behaviours. Women's health questionnaire - no significant difference but trend for prepared women to report fewer sexual problems. |
| Rothert 1997 | X |  |  | X |  | Across all groups, knowledge increased from time 1 to time 4. The change over time in knowledge was statistically significant. Knowledge level remained significantly greater than baseline, thus, the post intervention increase in knowledge was maintained over time. However, knowledge increased significantly less for participants in Group A compared to Groups B. The increase for groups B and C remained significantly greater than for Group A 12 months later. Knowledge increased more for Group B than for Group C between time 1 and time 2 and remained greater than for Group C at time 3 and time 4. Across experimental group, self-efficacy increased between time 1 and time 2, and remained higher than baseline at time 4. There were no significant differences between experimental groups. |
| Lemaire 1995 | X |  |  |  | X | Decrease in uncertainty statistically significant. Average scores on the 5-item true-false test of actual knowledge about menopause for 147 subjects increased. The differences were significant. |
| Patil 2022 | X |  |  |  |  | It shows that educational intervention was an effective for increase in knowledge. |
| Khandehroo 2022 |  |  | X | X |  | A significant difference was found between women of intervention versus control groups at follow-up, and in change from baseline to follow-up in all scores including MENQOL, health literacy, and self-efficacy. The quality of life was improved only in the intervention group. |

QoL: quality of life, MENQOL: menopause specific quality of life, FSFI: female sexual function index
